# Shifting the Paradigm: A Pilot Study Empowering Communities in Sri Lanka’s Dengue Prevention Efforts

**DOI:** 10.51894/001c.144108

**Published:** 2025-09-30

**Authors:** Inoka Manchanayake

**Affiliations:** 1 College of Osteopathic Medicine Michigan State University

## 31

### INTRODUCTION

Dengue virus is transmitted by the Aedes aegypti and A. albopictus mosquitoes. It is prevalent in tropical and subtropical regions worldwide. The incidence of dengue has increased in recent decades, spreading into non-endemic areas such as the United States and Europe. This expansion is linked to factors such as climate change, globalization, travel, trade, and urbanization, all of which facilitate the virus’s spread. While dengue can present with mild, non-specific symptoms, severe cases can lead to dengue hemorrhagic fever and shock syndrome, which can be fatal. According to the World Health Organization (WHO), the disease is endemic in over 100 countries. In Sri Lanka, a tropical island in the Indian Ocean, dengue is most prevalent in the Western province, including the densely populated area of Borella, located in the capital city of Colombo. The high incidence in this region is partly due to its diverse ethnic, cultural, religious, and socioeconomic makeup.

### OBJECTIVES

This pilot study aims to explore how Sri Lanka’s traditional “top-down” government-run dengue control program can be enhanced by incorporating a “bottom-up” strategy. This approach involves increasing community participation, leadership, and empowerment, to improve societal engagement, capacity building, and dengue prevention.

### METHODS

A three-week participatory observation study was conducted through fieldwork and house-to-house inspections with medical staff from the MOH Borella office. The study aimed to observe community values, behaviors, attitudes, and resistance to government-run dengue control programs. A sample of 300 households was selected to gather qualitative data. Following this, an intervention was introduced by creating a self-checklist for participants, which was tested in 30 randomly selected households.

### RESULTS

Anticipated results include improved community engagement, increased knowledge about dengue and resultant behavior change, reduction in dengue cases, more effective government programming that empowers local leadership, and the identification of barriers or resistance to dengue control programs. Results are anticipated by mid-March.

### CONCLUSION

This study recommends sustained mobilization of communities, enhancing societal participation through knowledge translation, and empowering individuals to seek and embrace local leadership in dengue prevention efforts.

